# TCR Redirected T Cells for Cancer Treatment: Achievements, Hurdles, and Goals

**DOI:** 10.3389/fimmu.2020.01689

**Published:** 2020-09-03

**Authors:** Francesco Manfredi, Beatrice Claudia Cianciotti, Alessia Potenza, Elena Tassi, Maddalena Noviello, Andrea Biondi, Fabio Ciceri, Chiara Bonini, Eliana Ruggiero

**Affiliations:** ^1^Vita-Salute San Raffaele University, Milan, Italy; ^2^Experimental Hematology Unit, Division of Immunology, Transplantation and Infectious Diseases, IRCCS San Raffaele Scientific Institute, Milan, Italy; ^3^Fondazione Centro San Raffaele, Milan, Italy; ^4^School of Medicine and Surgery, University of Milano – Bicocca, Milan, Italy; ^5^Clinica Pediatrica Università degli Studi di Milano Bicocca, Fondazione MBBM, Monza, Italy

**Keywords:** TCR - T cell receptor, genetic engineering, cancer immunotherapy, adoptive T cell immunotherapy, cancer immunoediting

## Abstract

Adoptive T cell therapy (ACT) is a rapidly evolving therapeutic approach designed to harness T cell specificity and function to fight diseases. Based on the evidence that T lymphocytes can mediate a potent anti-tumor response, initially ACT solely relied on the isolation, *in vitro* expansion, and infusion of tumor-infiltrating or circulating tumor-specific T cells. Although effective in a subset of cases, in the first ACT clinical trials several patients experienced disease progression, in some cases after temporary disease control. This evidence prompted researchers to improve ACT products by taking advantage of the continuously evolving gene engineering field and by improving manufacturing protocols, to enable the generation of effective and long-term persisting tumor-specific T cell products. Despite recent advances, several challenges, including prioritization of antigen targets, identification, and optimization of tumor-specific T cell receptors, in the development of tools enabling T cells to counteract the immunosuppressive tumor microenvironment, still need to be faced. This review aims at summarizing the major achievements, hurdles and possible solutions designed to improve the ACT efficacy and safety profile in the context of liquid and solid tumors.

## Introduction

**Figure 1:**
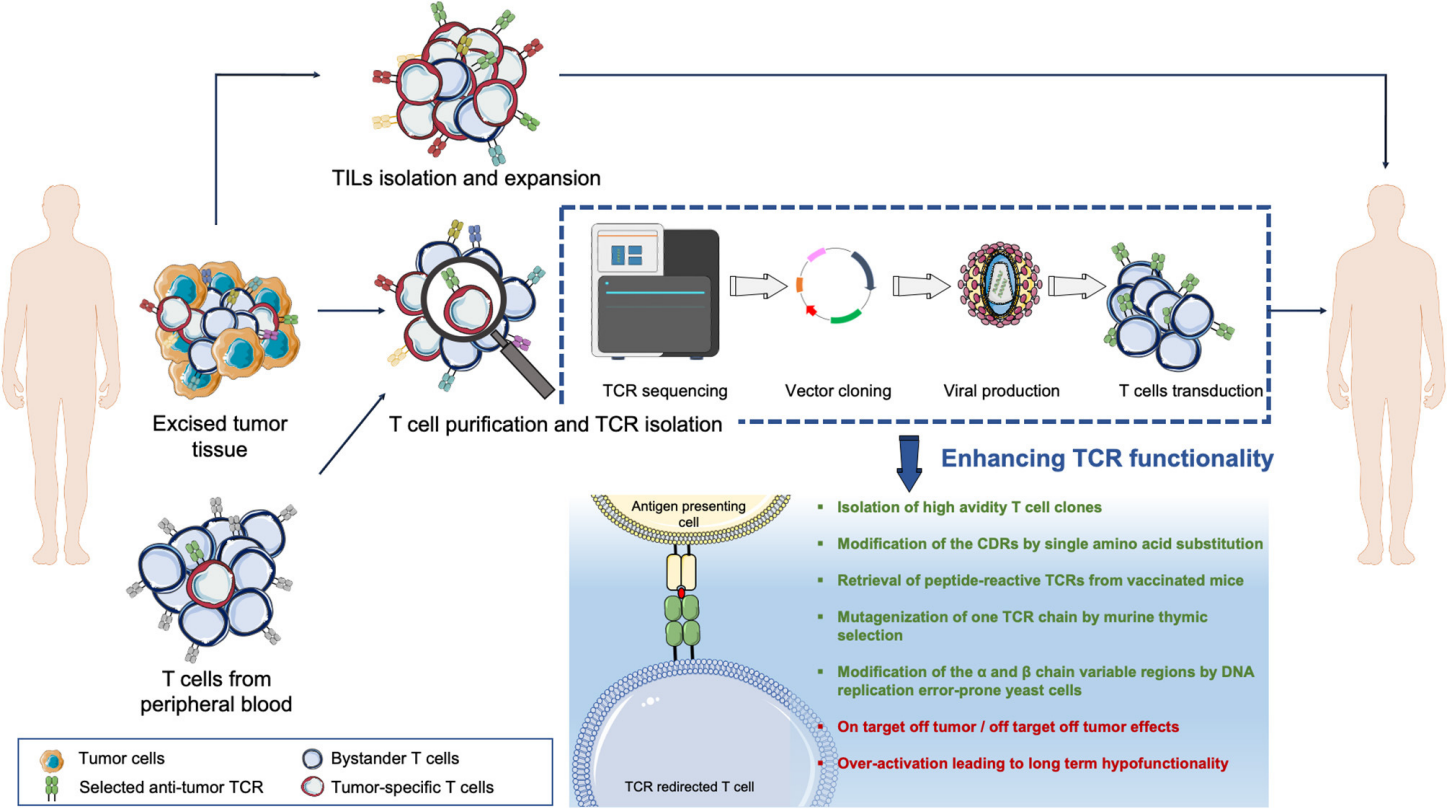
Overview of the TCR adoptive T cell therapy. Tumor-reactive lymphocytes can be isolated from either the tumor mass (tumor-infiltrating lymphocytes, TILs) or from the T cell pool circulating in patients' peripheral blood. T cells can be expanded *in vitro* and then re-infused back into the patient such as in TILs therapy. Else, the tumor-reactive T cell Receptor (TCR) genes can be isolated, sequenced, and transferred into acceptor T cells via vectors to redirect T cell specificities against tumor epitopes.

Adoptive T cell therapy for cancer (ACT) is a branch of cancer immunotherapy that relies on the ability to redirect T cell specificity to selectively target tumor antigens. ACT stemmed from two remarkable clinical observations: (i) The magnitude of T cells infiltrating tumor masses often correlates with response to treatment (1) and (ii) Allogeneic donor T cells infused in the context of hematopoietic stem cell transplantation promote clinical response in hematological malignancies (2). Initially, ACT solely relied on tumor-specific T cells isolated from the tumor masses and expanded *in vitro* (3). This approach was limited to resectable tumors from which enough T cells could be harvested and expanded. The development of gene engineering technologies dramatically changed the landscape of the ACT field, rapidly making this treatment accessible to an unprecedented number of patients and tumor types. By inserting an exogeneous T cell receptor (TCR) into cells, T cells specificity could be precisely redirected toward selected tumor antigens (Figure 1). This new opportunity shifted the research focus and raised some novel questions: the main issue was no more how to harvest a sufficient number of tumor-specific T cells from each single patient, but how to isolate and harness high-avidity tumor-specific TCRs, and how to proficiently generate and expand the most fit engineered T cells. The flexibility of the genetic modification tools offered the chance to insert and/or remove different genes in T cells and to permanently express, in the therapeutic products, entirely synthetic molecules. A striking deliverable produced by these efforts is represented by T cells expressing Chimeric Antigen Receptors (CARs), that generated astonishing clinical results against blood malignancies (4–9).

The outcomes of the first ACT clinical trials contributed to further elucidate the complex interplay between the immunosuppressive tumor microenvironment and the cellular players of immunity. Results suggested that modulation of *ex vivo* T cell expansion protocols and additional engineering of the T cell genome could be used to tweak T cell qualities, improving persistence and functionality of the therapeutic products. Several strategies were proposed to improve engineered T cell persistence, homing ability to the tumor site, capacity to recognize and eliminate tumor cells, and represent today's intense research lines. The possibility to modulate TCR affinity and T cell costimulatory and inhibitory signal pathways opens up novel therapeutic scenarios. The following review has the scope to summarize the cornerstones and the most relevant hurdles and efforts currently pursued to improve ACT.

## From Allogeneic Stem Cell Transplantation to Adoptive T Cell Therapy

Allogeneic Hematopoietic Stem cell transplantation (Allo-HSCT) is a therapeutic modality relying on the infusion of hematopoietic stem and progenitor cells, harvested from a healthy donor, to a patient previously conditioned with high-doses chemo-radiotherapy. Although initially developed to regenerate the bone marrow of patients with genetic diseases or with hematological malignancies requiring strong myeloablative chemotherapy (10, 11), Allo-HSCT proved able to control malignant cells largely through an immunological mechanism, as delineated by two major observations. Firstly, T-lymphocyte depleted grafts had a decreased efficacy in eradicating malignant diseases, suggesting that the donor to host immune response, and in particular the activity of allogeneic T cells, had *per se* an effect in abating the risk of relapse after transplant (2, 12, 13). Secondly, the infusion of circulating mature lymphocytes harvested from the donor (donor lymphocyte infusion, DLI) (14) correlated with the anti-leukemic effect (graft vs. leukemia, GvL) in a dose-dependent fashion (15). The efficacy of Allo-HSCT and DLI in restoring a state of disease remission represents one of the first compelling evidences of the potential of adoptive T cell therapy. Unfortunately, the benefits of allogeneic transplant and DLI against cancer are counterbalanced by toxicities, mainly due to the presence of a heterogeneous TCR repertoire with unknown specificities in the infused T cell population. Indeed, it's been calculated that ~10% of the T cell repertoire circulating in healthy donors is alloreactive (16). The most common manifestation of such toxicities is graft vs. host disease (GvHD), an immune reaction against the host's healthy tissues, occurring with varying degrees of severity but potentially fatal. The efforts to reduce toxicity while preserving the efficacy of DLI, and to export this therapeutic opportunity beyond the HSCT context, were the driving forces in promoting innovative ACT approaches.

The first ACT strategies tested with autologous T lymphocytes were based on the isolation of T cells infiltrating primary lesions resected from patients with melanoma (tumor-infiltrating lymphocytes, TILs), followed by their *in vitro* expansion with high-doses of interleukin-2 (IL-2) (17). The infusion of these cellular products, composed of an oligoclonal T cell repertoire incorporating CD4^+^ and CD8^+^ T cells, mediated potent anti-tumor responses with no toxicities in cell types other than melanocytes (3, 18, 19). The Objective Response Rate (ORR) observed was 41% across various clinical trials for patients with metastatic melanoma (20). Based on these encouraging results, the approach was widened and offered to patients affected by other solid tumors with variable outcomes, promising in some settings [e.g., sarcoma (21), cervical and ovarian cancer (22, 23)] but rather modest in others [e.g., renal (24), metastatic renal (25) and, colorectal (26, 27) cancer, Table 1]. The inconsistent efficacy of TILs may be linked to various causes: (i) the technical difficulties in isolating T cells from immune-cold tumors (44); (ii) the poor reactivity of the screened T cells, especially in tumors characterized by a low mutational burden (45, 46), and (iii) the overall low frequency of tumor-specific T cells infiltrating cancer lesions when compared to bystander T cells (47, 48). The high success rate of TILs therapy in melanoma can be in fact explained by the melanoma cells high tumor mutational burden, resulting in a heightened immunogenicity and a consequent enrichment of tumor-specific T lymphocytes (49).

**Table 1:** Overview of TCR-engineered T cell-based clinical trials.

**Disease**	**Epitope**	**Antigen**	**Antigen type**	**HLA restriction**	**Vector (nuclease)**	**Number of treated patients**	**ORR (%)**	**Infusion toxicities**	**References**
Melanoma	AAGIGILTV	MART-1	TAA (tissue restricted)	HLA-A*0201	Retrovirus	17	2 (12%)	none	(28)
Melanoma	AAGIGILTV	MART-1	TAA (tissue restricted)	HLA-A*0201	Retrovirus	20	6 (30%)	14 (skin rash), 11 (uveitis), 10 (hearing loss)	(29)
Melanoma	KTWGQYWQV	gp100	TAA (tissue restricted)	HLA-A*0201	Retrovirus	16	3 (19%)	15 (skin rash), 4 (uveitis), 5 (hearing loss)	(29)
Melanoma and synovial sarcoma	SLLMWITQC	NY-ESO-1	TAA (cancer/testis antigen)	HLA-A*0201	Retrovirus	11 and 6	5 (45%) and 4 (67%)	none	(30)
CRC metastatic and synovial sarcoma	IMIGVLVGV	CEA	TAA (tissue-restricted)	HLA-A*0201	Retrovirus	3	1 (33%)	3 (severe colitis)	(31)
Melanoma	EVDPIGHLY	MAGE-A3	TAA (Cancer/testis antigen)	HLA-A*01	Lentivirus	2	n.a.	2 (death due to cardiac toxicity)	(32)
Metastatic melanoma, sinovial sarcoma and esophageal cancer	KVAELVHF	MAGE-A3	TAA (Cancer/testis antigen)	HLA-A*0201	Lentivirus	7, 1 and 1	5 (56%)	2 (death), 2 (CNS symptoms)	(33)
Metastatic melanoma	EAAGIGILTV	MART-1	TAA (tissue restricted)	HLA-A*0201	Retrovirus	13	9 (69%)	2 (skin rash), 2 (CRS)	(34)
Esophageal cancer	KVAELVHF	MAGE-A4	TAA (cancer/testis antigen)	HLA-A*2402	Retrovirus	10	0 (0%)	None	(35)
Multiple Myeloma	SLLMWITQC	NY-ESO-1	TAA (cancer/testis antigen)	HLA-A*0201	Lentivirus	20	16 (80%)	None	(36)
Sarcoma plus myeloma	SLLMWITQC	NY-ESO-1	TAA (cancer/testis antigen)	HLA-A*0201	Retrovirus	18 and 20	11 (61%) and 11 (55%)	None	(37)
Leukemia	CMTWNQMNL	WT1	TAA (Transcription Factor)	HLA-A*2402	Retrovirus	8	2 (25%)	None	(38)
Metastatic synovial sarcoma	NY-ESO-1^c259^	NY-ESO-1	TAA (Cancer/testis antigen)	HLA-A*0201	Lentivirus	12	6 (50%)	11 (BM suppression)	(39)
Leukemia	RMFPNAPYL	WT1	TAA (Transcription Factor)	HLA-A*0201	Lentivirus	12	12 (100%)	9 (GvHD)	(40)
Synovial sarcoma, osteosarcoma, liposarcoma, peripheral malignant nerve sheet tumor	SLLMWITQC	NY-ESO-1	TAA (Cancer/testis antigen)	HLA-A*0201	Retrovirus	10	2 (20%)	1 (CRS)	(41)
Myeloma/liposarcoma	SLLMWITQC	NY-ESO-1	TAA (Cancer/testis antigen)	HLA-A*0201	Lentivirus	3	0 (0%)	None	(42)
					(CRISPR-Cas9)				
Synovial sarcoma	SLLMWITQC	NY-ESO-1	TAA (Cancer/testis antigen)	HLA-A*0201	Lentivirus	30	9 (30%)	n.a.	(43)

*BM, Bone Marrow; CEA, Carcino Embrionic Antigen; CNS, central nervous system; CRS, Cytokine Release Syndrome; gp100, glpycoprotein 100L; MAGE-A3/A4, Melanoma-Associated Antigen A3/A4; MART-1, Melanoma Antigen Recognized by T cells 1; NY-ESO-1, New York Esophageal Squamous Cell Carcinoma-1; ORR, Overall Response Rate; TAA, Tumor-Associated Antigen; GvHD, Graft vs. Host Disease; TCR, T cell Receptor; WT1, Wilms Tumor 1*.

To expand the beneficial effect of TILs while overcoming the hurdles intrinsically associated with this therapy, the use of circulating T cells, harvested from patients and stimulated *in vitro* with immunogenic cancer epitopes, was proposed. This approach promotes the selective expansion of the tumor-specific T cell fraction, in numbers sufficient to enable their re-infusion to patients, resulting in clinical benefits (50, 51). Nonetheless, the use of TILs and circulating T cells lead to the generation of a T cell population for which the affinity and functionality of the TCR could not be predicted *a priori* and whose ability to effectively induce clinical responses was tightly linked to the expansion potential of harvested cells.

Gene editing and gene transfer technologies greatly boosted the ACT field, allowing modification of the T cell genome and redirection of T lymphocytes specificities by inserting highly functional, tumor-specific TCRs (52) into patients' T cells, that could be subsequently expanded *in vitro*. In the 90s the discovery that the Fab region of an antibody could be efficiently fused to the CD3 zeta chain and to other costimulatory intracellular domains to create Chimeric Antigen Receptors (CARs), further revolutionized the T cell-based immunotherapy field. CARs are able to activate T cells upon binding to a surface receptor expressed by the target tumor cell (53) and their use proved instrumental in widening the therapeutic window of blood tumor treatment in otherwise poor survivors (4–9), thus confirming TCR-engineered T cells as a new therapeutic.

## Making Tumor Specific T Cells: From TCR Gene Transfer to TCR Gene Editing

The ability of T cells to respond to a wide spectrum of foreign antigens relies on the high variety of TCRs, heterodimeric glycoproteins composed of one α and one β chain associated to the CD3 complex (54), able to specifically interact with antigenic peptides bound to human leukocyte antigen (HLA) restriction elements (55). A series of genetic rearrangements in the α and β chain genes occur slightly differently in every single cell, thus creating a heterogeneous TCR repertoire that can recognize a vast epitope array. Hence, to fully characterize the T cell specificity it is necessary to determine the rearranged α and β chain sequences.

In the 80s, the progression of genomics allowed the isolation of TCR genes (56, 57) and the study in detail of their sequences. The advent of next generation sequencing technologies rendered feasible a comprehensive identification of tumor specific TCR sequences that are today used to genetically engineer T lymphocytes in adoptive T cell therapy studies.

### Gene Transfer at the Service of ACT

Most TCR-based gene therapy approaches rely on the *ex-vivo* transduction of T cells with viral vectors. The first vectors used in gene therapy were adenoviruses (58), vectors endowed with high cargo capacity (up to 30 kb) but unable to foster transgene integration in the host genome. This feature reduced the adenoviruses utility for ACT: since T cells robustly proliferate upon antigen encounters, integration of the transferred TCR genes in their genome is critical to the preservation of transgenic specificity in T cell progeny. Furthermore, the immunogenicity of adenoviral proteins, highlighted by the high incidence of adenovirus-specific neutralizing antibodies in humans, potentially leads to viral inactivation (59) or to life-threatening inflammatory responses (60), thus limiting their exploitation. Retroviral vectors (RV), instead, have been broadly used because of their wide cell tropism (61, 62), good integration capacity, and for the high and stable gene expression they convey. Cell division is required for RV transduction, but this limitation does not impact their use since T cells are highly proliferating *in vitro*. RV have been widely used to deliver a variety of molecules, including suicide genes (63–65), TCRs (28, 66), and CARs (53) in T lymphocytes. Lentiviral vectors (LV) gained interest more recently, particularly for their efficiency profile and their capacity for transducing dividing as well as non-dividing cells, a feature particularly relevant for the genetic manipulation of stem cells (67). The safety profile of RV and LV is guaranteed by a vector design ensuring replication incompetence (68, 69) and has been proven in human trials (70–72). Adeno-Associate Viruses (AAV) (73) have been widely used in cancer gene therapy and proven to be well-tolerated and safe. Still, the need to synthesize the complementarity strand to promote transgene integration represents a limitation. To circumvent the process, both strands can be packaged as a single molecule to pair and form a dsDNA as a self-complementary AAV vector (scAAV). While this technological advancement allowed AAV to be independent from host cell complementary strand synthesis (74), it almost halved the vector packaging capacity. Nonetheless, scAAV outperformed conventional AAVs in terms of efficacy in preclinical models (75, 76).

Integrating viral vectors insert the genetic cassette semi-randomly into the host genome, thus potentially leading to unwanted insertions in exons, that leads to disruption of the gene hit, or in enhancer regions, potentially altering gene regulation. Theoretically, viral integrations in oncogene regulatory elements or in tumor suppressor genes may contribute to oncogenic transformation (77), a rare event that, most importantly, has never been reported in engineered T cells. Nevertheless, several strategies have been implemented to increase the safety profile of integrating vectors. These include the elimination of viral genes responsible for virulence (78), splitting packaging genes into different plasmids (79), and the introduction of inactivation switches in the vectors constructs (80). In addition, chromatin insulator elements can be added to the flanking regions of the insertion cassette, acting as physical barriers to hamper the interactions between viral enhancers and other regulatory elements (81). To increase the safety profile of viral-mediated gene delivery, it is nowadays possible to instruct viral vectors to integrate into specific “safe harbors,” genomic regions distant from transcribed genes, enhancers, regulatory RNA, or microRNA regions to minimize the risk of perturbing gene expressions (82, 83). Identified safe harbors are either housekeeping genes, e.g., AAVS1 (84, 85) and ROSA26 (86), or specific loci not affecting gene expression and identified by mapping the viral vectors integration sites (87).

**Figure 2:**
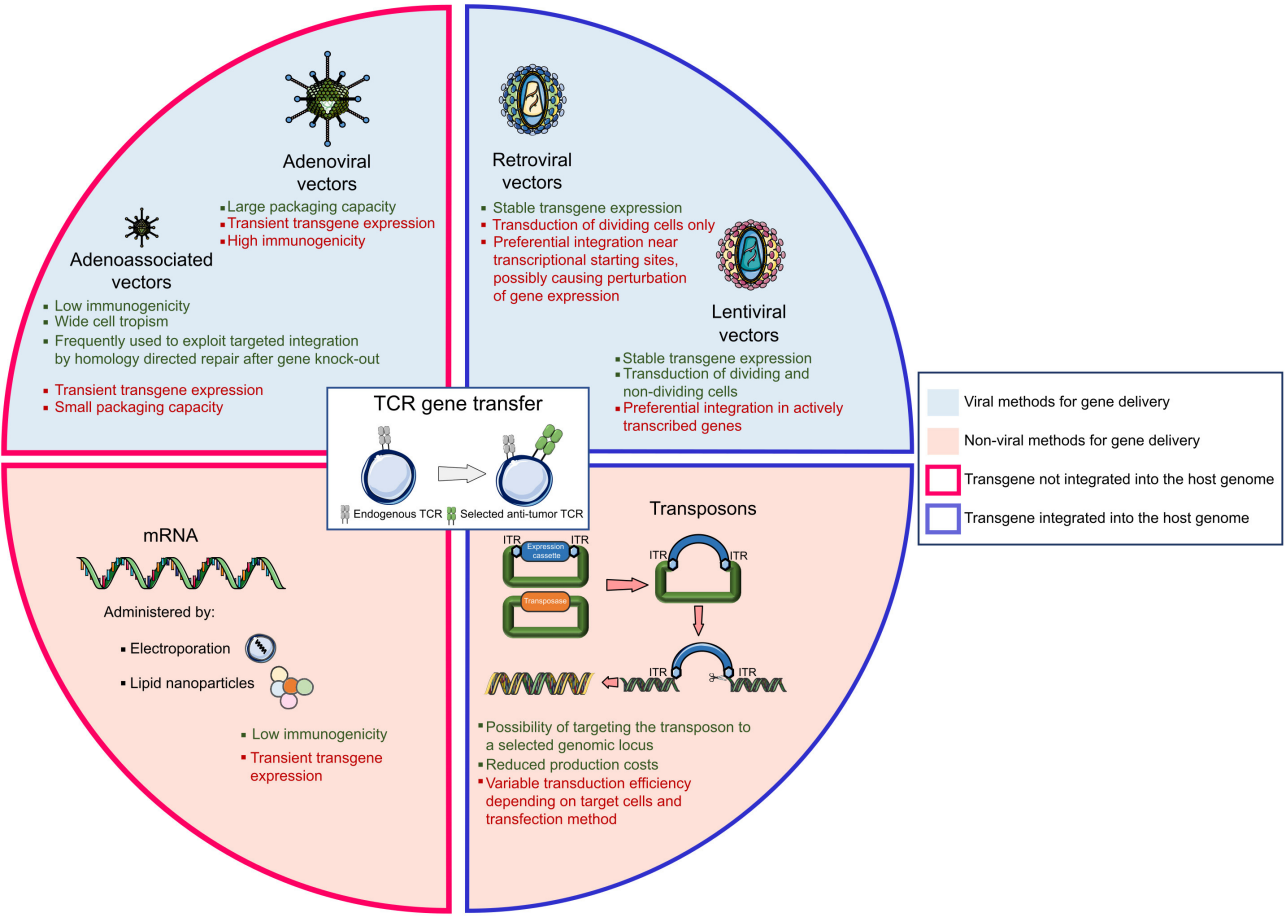
The landscape of gene delivery methods. The genetic transfer of an exogeneous T cell receptor (TCR) into a donor T cell can be obtained with different vectors, the most widely used being viral vectors, mRNA, and transposons systems. Strengths and weaknesses are listed for each technology.

In addition to viral vectors, a variety of non-viral gene transfer methods have been explored to transfer transgenes into T cells (Figure 2). Transposons are mobile elements composed of a transposase gene flanked by inverted terminal repeats (ITRs) (88). For the purpose of gene therapy, two plasmids are transfected together, one encoding for the transposase and the other one containing the expression cassette flanked by ITRs; upon entry, the transposase integrates the gene of interest in the genome. The so-called “Sleeping Beauty” transposon system gained the widest application, being able to transfer up to 6 kilobases into mammalian cells (89). This system may be considered as efficient as viral gene transfer, at least *in vitro*, if the transposon/transposase ratio is tightly controlled (90) to avoid the formation of functionally inactive transposase oligomers (91). In general, transient expression of the transposase is usually preferred, and ensured by mRNA electroporation (92). Transposons have an acceptable production cost and a low immunogenicity potential. Nonetheless, gene transfer efficiency varies according to the target cell and is sensitive to the size of the expression cassette. In the ACT field, transposons have been efficiently utilized to express functional CARs (9, 93–95) and TCRs (96–98) but their exploitation in clinical practice is still limited.

Messenger RNA-based gene transfer, usually achieved by mRNA electroporation or by enclosing the mRNA into lipid nanoparticles (99), is devoid of insertional mutagenesis risk. However, mRNA can convey only transient transgene expression and *in vitro* transcribed mRNAs could trigger cellular inflammatory reactions, whose incidence can be mitigated by introducing base modifications in the synthetic RNA (100). In ACT studies, CAR-T cells have been generated using mRNA gene transfer; still, multiple administrations of the engineered T cells were necessary to mediate tumor regression. Of interest, in a phase I clinical trial, CAR-T cells targeting mesothelin generated upon mRNA electroporation and administered to patients with advanced cancers proved safe and mediated anti-tumor activity despite transient persistence (101).

### TCR Gene Transfer

In 1986, a murine exogenous α and β TCR gene pair was successfully transferred into another cytotoxic T cell, endowing the recipient cell with a new TCR specificity (52). The efficacy of the TCR gene transfer was tested in immortalized T cells, where the cDNA of a MART-1-specific TCR isolated from melanoma TILs was stably expressed (102) and, shortly after, on primary human T cells, granting recipient T cells cytolytic activities specifically toward their target epitope. The encouraging safety and efficacy pre-clinical results observed by targeting MART-1 (103, 104), the murine MDM2 oncoprotein (105) and the EBV-associated LMP protein (106), prompted the approval of TCR gene transfer in human clinical trials. In the context of metastatic melanoma, TCR-transferred T cells successfully induced tumor regression in two out of 15 patients and persisted *in vivo* for at least 2 months after infusion (28). The safety profile of TCR-transferred T cells specific for MART-1 or gp100 was similar to that of TILs, with on-target off-tumor toxicities toward skin and eye melanocytes (107). Thanks to these seminal results, the TCR transfer clinical application was widened to other relevant targets, such as the New York esophageal squamous cell carcinoma (NY-ESO)-1, expressed in Melanoma and Synovial Sarcoma (30, 43) and the carcinoembryonic antigen (CEA), expressed in colorectal cancer (31).

The broader use of TCR transfer, however, underlined some of the limitations of this new technology. Firstly, endogenous and exogenous TCRs competed for assembly with the CD3 subunits (108), thus resulting in suboptimal surface expression of the transferred receptor. Secondly, α and β chains from exogenous and endogenous TCRs could mis-pair, further diluting the expression of the correctly paired tumor-specific receptor and introducing new specificities, potentially leading to unwanted toxic reactivities (109). TCR mispairing has been described *in vitro* by using human cells (109) and was associated to immune-mediated toxicities in murine models (110). So far, no events potentially associated to TCR mispairings have been reported in clinical trials. Nonetheless, to address this safety issue and to increase the expression level of the exogenous TCR, several strategies have been proposed: (i) the replacement of the human TCR constant region, essential for pairing, with a murine-derived sequence (111), (ii) the introduction of cysteine residues to stabilize proper pairing of the TCR chains via disulphide bonds (112, 113), (iii) the generation of a human TCR incorporating the CD3ζ chain (114), (iv) the swapping of TCR constant domains between the α and β chains (115), and (v) the incorporation in the vector cassette of small interfering RNA sequences able to reduce the expression of the endogenous TCR genes (116). To overcome the limitations of TCR gene transfer, nascent genome editing technology has been exploited to develop the TCR gene editing approach (117).

### Genome Editing in the Service of ACT

The use of artificially modified nucleases enables the disruption of the genes encoding α and β chains of the endogenous TCR, thus completely and permanently avoiding the risk of TCR mispairing and the mutual dilution effect resulting from the expression of four TCR chains in a single cell. Artificial nucleases bind DNA in selected genomic regions, in which they mediate a DNA double-stranded break (DSB), either repaired by the high-fidelity homologous direct repair (HDR) system or by the mutagenic non-homologous end joining repair machinery (NHEJ). HDR uses a DNA template, usually the sister allele, to correct the break and restore gene function, while NHEJ introduces or erases a variable number of nucleotides upon repair, with the chance of creating premature stop codons and frameshift mutations. Both repair mechanisms can be exploited for gene therapy purposes, with different aims: HDR is suitable for gene correction when an exogenous donor DNA template is delivered with the nuclease (118), while NHEJ is preferred if a gene has to be disrupted (119).

The zinc fingers nucleases (ZFNs), among the first efficient gene editing tools developed, are large multimeric molecules, each monomer targeting a 3-4 DNA base pair sequence, linked to the FokI endonuclease (120). While the multimers confer ZFNs specificity, that can be increased even by elongating the length of the multimers, the endonuclease mediates DNA cleavage. This gene editing tool supported the first genome editing clinical applications (121) and the first TCR gene editing approach. In fact, ZFN-mediated disruption of the endogenous TCR has been combined with LV gene transfer to efficiently generate WT1-specific TCR-edited T cells that outperformed TCR gene transferred T cells in safety, specificity, and efficacy *in vitro* and *in vivo* (117). Despite these encouraging results, the first protocol reported required 40 days of manufacturing to be completed and multiple manipulation steps. To improve feasibility, a single-editing strategy, based on the sole disruption of the TCR α chain gene was proposed resulting in optimal expression of a NY-ESO-1-specific TCR and efficient tumor rejection in animal models, in the absence of adverse events (122).

An alternative to the ZFNs system is represented by transcription activator-like effector nucleases (TALENs), small (33–35 amino acids) transcription factors fused with an endonuclease domain (123). TALENs specificity is modified by mutating the two hyper-variable residues that bind the DNA helix. The nucleotide sequence recognized by TALENs is fairly short, increasing the likelihood of off-target binding sites throughout the genome and potentially leading to unwanted DNA breaks. To overcome this limitation, the DNA binding regions can be elongated by multiplying the hyper-variable residues, hence increasing TALENs specificity at the expense of a more complicated protein design. In the ACT context, TALENs have been proficiently used to disrupt endogenous TCR genes in preclinical models and in clinical trials (124, 125).

Meganucleases represent alternative genome editing tools originating from naturally occurring endonucleases that directly bind DNA (126). Meganucleases present some advantages, such as the generation of a 3′overhang at the cleavage site that favors HDR when compared with 5′overhang, and their overall small size, suitable for several delivery methods (127). Still, the difficulty in separating the endonuclease cleavage domains from the DNA binding site limits the number of DNA sequences that can be targeted. To circumvent this obstacle, chimeric proteins have been generated by fusing meganucleases with ZFNs and/or TALENs DNA binding domains, at the expense of increased manufacturing complexity (128, 129).

The introduction of the CRISPR/Cas9 nucleases, bacterial proteins adapted to excise phage DNA fragments (130), completely revolutionized the genome editing field. While ZFNs and TALENs recognize the target DNA sequence via protein-DNA interaction, the CRISPR/Cas9 system relies on a short RNA sequence (single guide RNA, sgRNA). The RNA interacts with the Cas9, conferring the binding specificity and guiding the nuclease activity (131). The CRISPR/Cas9 platform is highly efficient and versatile, since the specific DNA binding is entirely mediated by the sgRNA, short enough to be easily synthetized *in vitro* but long enough to ensure high specificity. Compared to the previously developed nucleases, the CRISPR/Cas9 system provides three major advantages: (i) rapid and relatively inexpensive manufacturing, (ii) the possibility of multiplex genome engineering obtained by simultaneously targeting several genes, and (iii) compliance with several delivery systems adapted to different cell types (132).

Multiplex genome engineering is a remarkable feature of CRISPR/Cas9, not easily achieved with other nucleases. The possibility of disrupting genes in a single step streamlined different editing procedures (133) and had a direct impact on ACT manufacturing processes, where the synchronous disruption of the α and β TCR chains (42, 134) can sensibly decrease the *in vitro* manipulation time. In addition, a template strand can be delivered together with the CRISPR/Cas9 system, allowing the integration of the genetic material exactly at the cleavage site (135).

Different tools can be employed to deliver CRISPR/Cas9 complexes into cells. Plasmid delivery has been used (136), but with suboptimal efficiency and with an increased risk of plasmid integration in the host genome. Furthermore, the expression of the Cas9 protein is retained for a fairly long amount of time, increasing the likelihood of adverse immune responses or off-target gene editing. An alternative approach depends on delivering the sgRNA together with the *in vitro* transcribed Cas9 mRNA (137), ensuring transient Cas9 expression but posing the risk of decreased cleavage efficiency. Lastly, the native Cas9 protein can be pre-assembled *in vitro* with sgRNA in a ribonucleoprotein complex and then electroporated into the target cells (138). This transfer method overcomes the need for transcription/translation and the risk of intracellular degradation of the free sgRNA (139), thus improving safety and reducing off-target mutagenesis risks.

As for ZFNs (140–142) and TALENs (143, 144), a side effect of the CRISPR/Cas9 system is the risk of editing off-target genes. The nuclease activity can potentially cause DNA strand breaks in other genomic regions, knocking-down unwanted genes or promoting genome translocations (145, 146). Off-target editing can also affect the RNA transcriptome, with toxic consequences for the cell (147). Three methods have been employed to minimize off-targets while increasing on-target activity when using CRISPR/Cas9: (i) the use of modified sgRNAs with higher specificity for the target site, (ii) the titration of the sgRNA and Cas9 ratio (148), and (iii) the introduction of a single point mutation in the Cas9 (149). Furthermore, additional enzymes have been incorporated in the system to mediate base editing without affecting the transcriptome (150).

Several techniques have recently been optimized to map off-target cleavage sites. Mutation detection assays using T7 endonuclease followed by deep sequencing of the resulting amplicons have been initially used, but their sensitivity is limited, especially when dealing with large deletions (151). The tendency of integrase-defective lentiviral vectors to incorporate into DSBs can be exploited to barcode regions of Cas9 activity (152). BLESS [direct *in situ* Breaks Labeling, Enrichment on Streptavidin and next-generation Sequencing (153)] can map double-strand breaks by using biotinylated linkers that are incorporated at the DSB site; the biotinylated DNA regions are then purified and the captured DNA fragments sequenced. One of the most sensitive off-target detection assay is GUIDE-seq (genome-wide, unbiased identification of DSBs enabled by sequencing), where short phosphorylated double-stranded oligo-deoxynucleotides are incorporated into DSBs to detect Cas9 cleavage sites (154). More rarely, nucleases can cause chromosomal translocation that can be detected using high-throughput, genome-wide translocation sequencing (HTGTS) methods (155), chromatin immunoprecipitation sequencing (ChIP-seq) (156), and digenome-seq or the recently proposed CIRCLE-Seq (157).

**Figure 3:**
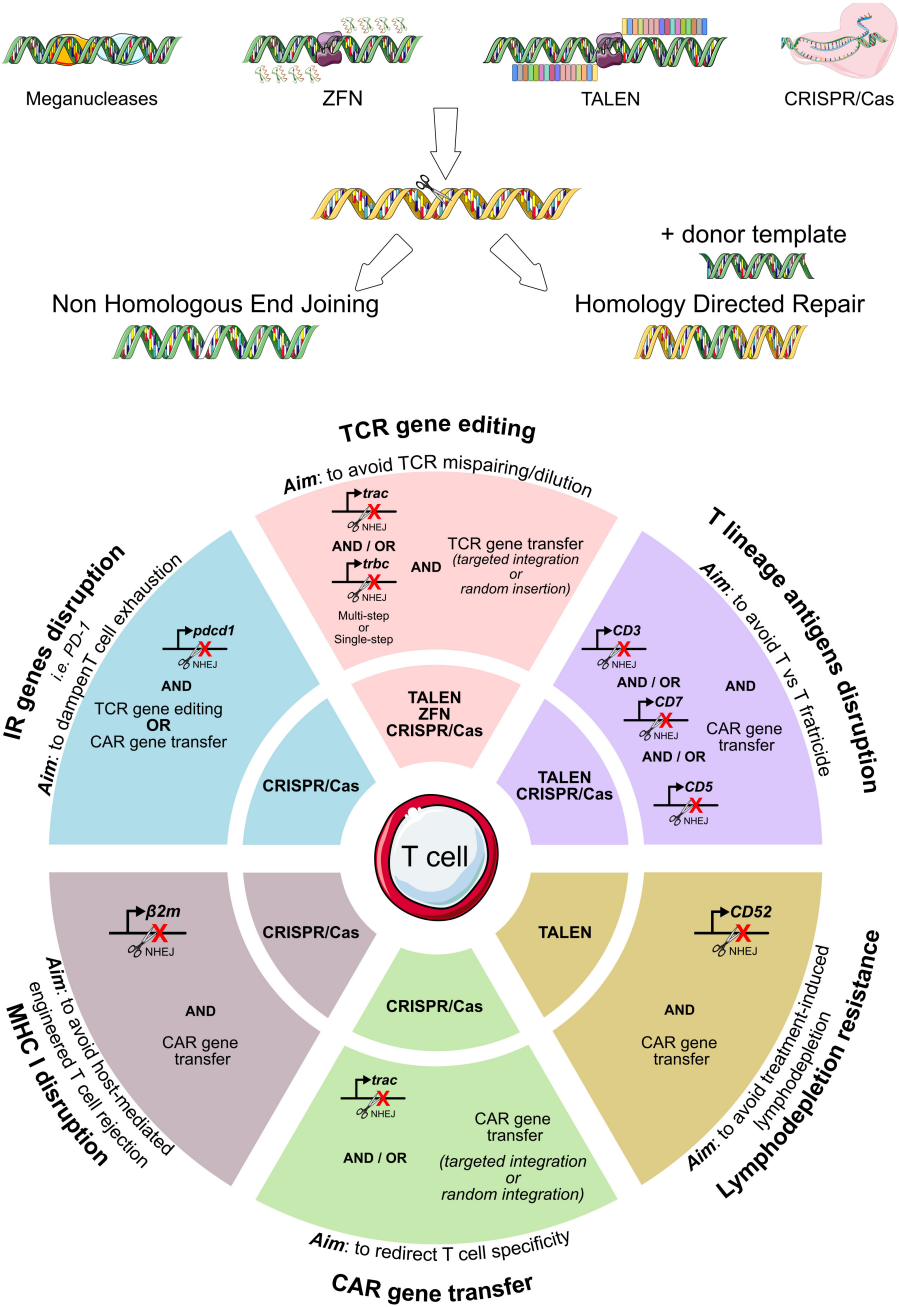
Genome editing exploitation for adoptive T cell therapy. To eliminate the expression of T cell genes, meganucleases, transcription activator-like effector nucleases (TALENs), and zinc-finger nucleases (ZFNs), the CRISPR/Cas9 system can be employed. A summary of the genes edited in the context of adoptive T cell therapy (TCR- or CAR-T cell immunotherapies) is reported, together with the specific nuclease system used. CAR, chimeric antigen receptor; TCR, T cell receptor.

All described genome editing technologies have been employed for the modification of either hematopoietic stem cells or T cells. As summarized in Figure 3, genome editing has been exploited with several purposes in the ACT field: to completely redirect T cell specificity (42, 117, 124, 158–160), to avoid the risk of GvHD or fratricide effects mediated by CAR-T cells (161–165), to make adoptively transferred T cells resistant to the immunosuppressive environment (42, 166–173) or to lymphodepleting drugs (174, 175). The high efficiency of gene editing and the overall flexibility of the CRISPR/Cas9 system makes it the most relevant tool to precisely and rapidly edit high numbers of T cells to be used in ACT. Nonetheless, issues remain to be addressed regarding the manufacturing, the delivery, and the broad accessibility of genome editing products to patients (176).

## Antigen Identification and TCR Gene Hunting

### Different Classes of Target Antigens

One of the major questions in today's adoptive T cell gene therapy is the choice of the target antigen. Theoretically, the ideal candidate should be (i) expressed on tumor cells and not on healthy tissues (to avoid toxicities), (ii) expressed on cancer stem cells (to promote tumor eradication), (iii) associated with the oncogenic process (to reduce the risks of tumor immune evasion), (iv) able to elicit an immune response, and (v) efficiently processed and presented in the context of a common HLA allele (177). Unlike CAR-T cells, TCR-redirected T cells can target antigens independently of their intracellular localization, as soon as they are processed and presented by HLA molecules. For the majority of cancer types, the ideal antigen is yet to be identified, and the search is proving more difficult than expected, particularly for those tumors with undefined clonal evolution and not fully understood in terms of molecular pathogenesis.

The two major classes of antigens in the context of ACT are tumor-associated antigens (TAAs) and neoantigens. TAAs are epitopes originated from endogenous wild-type proteins whose expression is increased in tumors and limited in magnitude or in spatial expression in healthy tissues; neoantigens are instead epitopes derived from somatic DNA alterations.

Different TAAs have been investigated for their potential therapeutic relevance (178): cancer/testis antigens such as melanoma-associated antigen (MAGE)-A3 (179, 180), MAGE-A4 (35, 181), and New York esophageal squamous cell carcinoma (NY-ESO)-1 (182); oncogenes/oncosuppressors such as WT1 (50) and p53 (183); tissue-restricted/differentiation antigens such as MART-1 (28, 34), gp100 (29), or CEA (31). The toxicity and safety profile of T cell therapies targeting TAAs seem to be heterogeneous, depending on the chosen epitope. Several TAA-specific TCRs showed important side effects: cardiovascular and neurological toxicities [with MAGE-3 specific T cells (32, 33)], undesired recognition of melanocytes (with MART-1 specific T cells) (28), and severe transient colitis (with CEA specific T cells) (31). TCR targeting NY-ESO-1, instead, conveyed no toxicities but limited clinical response in a small cohort of patients affected by melanoma and synovial sarcoma (30) and of sarcomas/nerve sheet tumors (41). Its application appeared promising in various pre-clinical tumor models, both in terms of efficacy and safety [bladder (184), ovarian (185), esophageal (186) and prostate cancers (187), multiple myeloma (188), medulloblastoma (189), non-small cell lung carcinoma (190) mesenchymal tumors (191), and breast cancer (192)] but clinical studies are needed to validate these results. The expected good toxicity profile may reside in the limited NY-ESO-1 expression in healthy tissues, essentially restricted to the gonads, an immune-privileged site.

Focusing on neoantigens appears to be another efficient choice for cancer immunotherapy (193). Neoantigens represent the main target of autologous T cell responses in patients treated with TILs (194). Since neoantigens peptides are not presented to thymocytes, T cells specific for those epitopes are not deleted by central tolerance mechanisms, thus the chance of retrieving high affinity TCRs is enhanced. However, the clinical exploitation of neoantigens in TCR-mediated ACT is hindered by their intrinsic qualities: (i) neoantigen-forming mutations tend to differ among patients, making difficult the development of a widely applicable immunotherapeutic product, and (ii) neoantigen expression might be heterogeneous across the tumor tissue. This is particularly true for passenger mutations, random alterations caused by genome instability and not homogeneously spread in the tumor mass (45). In addition, the mutational rate is highly variable in different tumors. In fact, the likelihood of identifying neoantigens from tumors with a low mutational load is poor and their relevance as therapeutic targets is limited (195). Despite these limitations, initial reports highlighting the occurrence of immunogenic neoantigens widely shared in tumor cells and among patients are emerging (196). Additionally, thanks to their broad expression in cancer cells and to their involvement in oncogenesis, founder mutations may be considered promising ACT candidate targets (197). However, epitopes arising from founder mutations may be poorly immunogenic or differ among patients, thus reducing their appeal.

Overall, the interest in neoantigens has increased in recent years, and more than 100 clinical trials exploiting these candidates are currently in progress. However, the ongoing trials largely rely on *in vivo* peptide vaccination rather than on engineered T cell infusions, further underlining the difficulties in validating a proper target for adoptive T cell therapy (198).

A third possible choice of targets is represented by Minor Histocompatibility antigens (MiHA), peptides derived by polymorphic intracellular proteins, potentially overexpressed by tumor cells. The most known examples are HA-1 and HB-1 (199–201), expressed selectively by hematopoietic cells and by different liquid cancers, thus constituting highly relevant targets (202, 203). Since MiHA derive from coding regions of polymorphic genomic sites, coupling HA-1-directed T cell therapy with Allo-HSCT from a recipient not harboring the same single nucleotide polymorphism (SNP) could provide a valuable therapeutic strategy, able to spare the donor hematopoietic stem cell population while eradicating cancer. So far, HA-1-specific engineered T cells have been tested *in vitro* and in preclinical models, showing an optimal efficacy and safety profile (204). However, MiHA targeting requires specific combination of SNPs and HLA allele between the donor and recipients, which reduces the broad applicability of this approach.

A fourth source of antigens are proteins encoded by oncogenic viruses. Being involved in tumorigenesis, viral epitopes are shared uniformly by the tumor mass and, due to their nature of foreign molecules, they're potentially highly immunogenic. Hereby, the isolation of high-avidity viral-specific T cells is easier than with other antigen classes, making these targets particularly appealing for ACT. Unfortunately, few tumors have a clear viral pathogenesis, thus this antigen source is currently mostly limited to some HPV and EBV-associated malignancies (205, 206).

### The Challenges in Selecting Tumor Antigens and Tumor-Specific TCRs

Apart from choosing the antigen class of interest, tumor screening for immunologically relevant epitopes is a particularly laborious process. The most used and standardized strategy relies on paired whole-exome sequencing or, alternatively, the comparative RNA sequencing analysis of tumor and of healthy tissues to determine differently expressed genes. Once the most promising hits are identified, *in silico* assessment of HLA presentation and binding (207) is required. In this regard, prediction algorithms are still suboptimal (208), not always accurate and they very often lead to false positive results (209). Thus, extensive validation of the results is always required. The analysis of the tumor ligandome can now be considered a good alternative approach. This technology is based on mass spectrometry typing of all the peptides eluted from the HLAs of a specific tumor type or tumor cell line (210). Here too, the amino-acidic sequences retrieved need to be validated for their relevance in the tumor setting. Despite the great potential of this methodology, some limitations still remain, namely the high number of tumor cells needed, an endpoint is not always attainable when using primary tumor samples, and the low number of epitopes retrieved upon *in silico* and *in vitro* screening (211, 212).

Finding the perfect immunogenic epitope is only half of the issue, since the typing of epitope reactive TCRs may prove challenging as well. To isolate a tumor-reactive T cell, a proper source must be selected, the clone of interest must ideally harbor a high avidity TCR and reach a significant level of frequency and purity. These prerequisites are fundamental for the successful retrieval of functional TCR αβ nucleotide sequences.

T cell isolation is particularly challenging especially for poorly immunogenic tumors and for those in which the T cell infiltrate is scant. Recent works showed that, in different cancers, tumor-reactive TILs represent only a minimal fraction of the total T cell subpopulation infiltrating the tumor (47) and that reinvigoration of tumor immunity is associated with recruitment of new T cell clones (213). These observation lead to the hypothesis that selecting T cell clones on the basis of their abundance in the tumor may be misleading. In addition, the poor efficiency of the T cell *ex vivo* expansion procedures may be taken in consideration as a limiting factor in the retrieval of tumor-specific T cells, especially when studying lymphocytes originated from tumors characterized by a microenvironment known to blunt T cell proliferation (214).

When tumor specific T cells are retrieved, the greatest challenge of the TCR sequencing step is the correct pairing of the cancer-specific α and β TCR chains for each T cell clone. The initial approach for the identification of the TCR repertoire was based on single cell cloning coupled with Sanger sequencing (215, 216), which makes it difficult to estimate the overall repertoire diversity. The field greatly benefited from the introduction of high-throughput sequencing technologies which enabled researchers to profile the diversity of millions of TCR molecules in the analyzed samples. With this approach a complete overview of the TCR sequences constituting the repertoire of the sample is obtained (217–219). However, to successfully characterize and select the TCR αβ pair of interest among the numerous sequences retrieved, the specimen needs to be enriched in anti-tumor specificities in order to obtain an oligoclonal population. To this aim sequencing can be preceded by enriching steps, such as co-culturing TILs with tumor cells harvested *ex vivo* or performing serial stimulations with professional antigen-presenting cells pulsed with the peptide (or peptide library) of interest. In the latter case, an entire protein sequence can be screened by epitope scanning (220).

An interesting approach that avoids the *ex vivo* enrichment step is PAIRseq: the sample is split into parallel PCR runs, each one tagged with a specific barcode, and then the results are deconvoluted to identify proper TCR pairs (221). An evolution of this laborious setting is perhaps single cell RNA sequencing, where the use of a cell-specific oligo-DNA barcode allows researchers to retrieve TCRs at single cell resolution (222). The typical output of these systems is generally hundreds or thousands of TCR pairs, questioning whether or not faster sequencing translates to a more laborious validation phase. Alternatively, recent advances in the proteogenomic field may speed up the selection of tumor-specific TCRs. In fact, it is now possible to combine single-cell resolution TCR sequencing with barcoded multimers loaded with a specific HLA molecule and with a selected tumor epitope (222). This approach is extremely helpful in characterizing, in a single step, both the tumor specific TCR sequence and its epitope specificity. This new technology may greatly speed up the isolation of TCRs directly from human samples, avoiding any enriching steps and jumping directly to the functional validation of newly retrieved TCR sequences *in vitro*.

Another point of discussion is the choice of the ideal specimen to be used for the isolation of tumor-reactive T cell clones. Hunting for tumor-specific T cell receptors directly from the tumor site in patients has historically been the most straightforward choice (17) since anti-tumor reactivities can be intuitively more abundant in the tumor mass. However, the development of a tumor implies an escape from immune surveillance, suggesting either that the infiltrating T cells present at the tumor site were not efficient enough to eradicate the disease, thus questioning their use, or that these highly specific cells were blunted in their activity by the tumor microenvironment (223). In the latter case, the anti-tumor efficacy of the tumor-specific T lymphocytes assessed by *in vitro* functional assays upon isolation may not be informative, but the TCR is worth isolating and employing in TCR transfer approaches. Recent reports (224, 225) also demonstrated that the exhaustion signature could be exploited, defining a T cell subset enriched with neoantigen-specific T cells.

Since tumor-reactive T cells also circulate throughout the body, patients' peripheral blood and lymph nodes from tumor patients are suitable sites to harvest these cells, and often the only available sites for tumors that cannot be excised. Tumor-specific T cells have been found in tumor-draining lymph nodes, and efficiently used for ACT (226). Melanoma-specific T cells have been enriched and expanded *ex vivo* starting from peripheral blood (227). However, at least in some tumor types, the low frequency of circulating tumor-reactive T cell clones (228) might impair their retrieval.

### Trading Toxicity With Efficacy

In the process of hunting for new TCR specificities, the aim is to define the TCR sequences most efficiently mediating tumor lytic functions. These highly promising TCRs can be collected and used for off-the-shelf immunotherapeutic approaches readily accessible for each candidate patient. The leading TCRs are the ones with the highest binding affinity and avidity (229) toward the HLA-peptide complex, the fastest association rate and the slowest dissociation speed (230, 231). According to this concept, isolated TCRs were screened for strong and fast killing efficacy and the most suitable ones were further developed. In addition, TCRs were modified in their complementary-determining regions (CDRs) *in vitro* to artificially increase their affinity for the target, thus overcoming the barrier of thymic selection, that deletes thymocytes harboring autoreactive high avidity TCRs. Several approaches have been recently exploited with the ultimate aim of generating high affinity TCRs: (i) mutations in complementarity determining regions by sequential single amino acid substitutions (232–234), (ii) vaccination of mice and consequent retrieval of peptide-reactive TCRs (235), (iii) murine thymic selection to mutagenize one of the TCR chains (236), (iv) transfer of the entire human TCR αβ gene loci into mice to educate T cells against human self-antigens (237, 238), and (v) DNA replication error-prone yeast cells to modify the α and β chain variable regions (239). These techniques have been particularly useful when dealing with TAAs, since highly avid T cell clones recognizing these antigens in a specific and efficient manner have been difficult to isolate.

The advantages of TCR affinity enhancement have been demonstrated *in vitro* (240–242) and in clinical trials (30, 36, 37, 39). The opposite side of the coin, though, is the risk of enabling engineered T cells to respond to tissues displaying low antigen expression, fostering on- or off-target off-tumor toxicities (243). A clinical trial with an artificially enhanced TCR directed against MAGE-A3 proved highly efficient in eradicating tumor cells but was also endowed with a remarkable off-target off-tumor cardiac toxicity (32, 244), leading to the suspension of the trial. On the same line, an artificially-enhanced TCR directed against CEA was associated with the occurrence of on-target off-tumor reactions and strong systemic inflammation, underlining the limitation of procedures aimed at enhancing TCR avidity when targeting TAAs (31). As a matter of fact, it proved very challenging to predict any possible cross-reactivity of engineered T cells against human tissues. The most commonly used techniques, *in vitro* testing and epitope alanine scanning, have been further refined in recent years (241, 244) and extended to scan all the possible amino acid substitutions in the target epitope (245). Nevertheless, concerns about affinity enhancing techniques still persist. In this context it might prove safe to introduce a kill switch in engineered T cells, enabling their ablation if necessary (63, 246). Otherwise, the conditioning regimen prior to T cell infusion can be modulated, reducing therapy-induced tissue damage and antigen spreading, two phenomena potentially fostering off-target reactions.

In addition to the adverse events observed in clinical trials, the modification of TCR affinity may also convey excessive activation signals to T cells, that could lead to hypo-functionality and/or premature T cell death. An extensive and continuous activation is indeed detrimental for T cell function (247–249). Reports in the context of TCR engineering are still scant (40) but new insights from the field of CAR-T cell therapy have highlighted this issue (250). Since TCRs is even more sensitive to antigen density variations than CARs (251), it's reasonable to suppose that this mechanism could be relevant in the context of TCR engineering. The picture might even be more complex with TCRs, since costimulatory signals are more tightly involved in the immunological synapse (252) than in CAR-T cells, a feature that provides more flexibility but that requires greater attention to signal tuning.

## Persistence of Adoptively Transferred T Cells and Clinical Responses

Nowadays, it's still unclear which variables impact long-term persistence in adoptively transferred T cells the most. This scientific question is particularly relevant because T cell persistence is a fundamental requisite for durable immunosurveillance. Whether immunosurveillance is required for the maintenance of clinical remission is still a matter of debate. However, reports indicate that the sustained and prolonged *in vivo* expansion of engineered T cells correlates with relapse-free survival and tumor control (39, 40). Several measures have been implemented to foster ACT persistence, including the use of preconditioning regimens and the choice of manufacturing protocols able to enrich memory cells. The use of a lymphodepleting conditioning regimen prior to ACT inhibits host immune cells, including regulatory T cells (T_regs_), and favors the accumulation of homeostatic cytokines (253), critical in sustaining engineered T cell engraftment and expansion (254, 255).

The administration of low-doses IL-2 can also sustain adoptively transferred T cell proliferation *in vivo* (17, 19) and has been included in several ACT protocols. The use of IL-2, however, conveys the risk of toxic reactions related to the activation of bystander host cells. Furthermore, prolonged administration of this cytokine was shown to preferentially expand T_regs_ (256).

The cellular composition, in terms of subsets and differentiation of the therapeutic product, has a direct impact on efficacy. Long telomeres (257), CD27, and CD28 co-expression (258) on TILs were associated to clinical responses in initial ACT trials. With engineered T cell products, the co-infusion of CD4 and CD8 cells fosters T cell persistence (8). In some ACT applications, manufacturing includes a selection step to enrich the product in CD8 central memory (T_CM_) lymphocytes (259). The polyfunctionality of adoptively transferred engineered T cells correlated with clinical responses in several clinical trials, targeting NY-ESO-1 (36), MART-1 (29), and WT1 (38). The relevance of the intrinsic qualities of the infused T cells was further highlighted by the observation that even low numbers (10^5^) of highly fit engineered T cells were sufficient to mediate anti-tumor responses (260, 261). Based on these observations and with the final aim of improving the fitness of the infused T cell products, different T cell expansion protocols have been developed and compared. T cell activation with phytohaemagglutinin (PHA) was shown to promote T cell expansion, but also T cell terminal differentiation (262), whereas stimulation with an anti-CD3 monoclonal antibody coupled with high doses of IL-2 reduced the TCR repertoire diversity and enhanced apoptosis (263). The combination of TCR triggering with co-stimulation, obtained thanks to the use of anti-CD3 and anti-CD28 antibodies, followed by the culture of the T cells in the presence of high-doses of IL-2, improved the fitness of the cellular products (264).

Despite showing potent tumor killing abilities *in vitro*, effector T cells were paradoxically less effective than early differentiated T cells when transferred in tumor-bearing mice (265). These results can be explained by the progressive model of mature T lymphocyte differentiation (266). Upon antigen encounter naïve T cells differentiate into stem cells memory T cells (T_SCM_), T_CM_ and subsequently in effector memory and terminally differentiated cells, progressively losing proliferating and persistence ability. Recent studies demonstrated that T_SCM_, originating directly from naïve T cells (267, 268), are endowed with stem cell-like properties and with the ability to persist for decades *in vivo* (269, 270). The persistence capacity of T_SCM_ was confirmed in patients treated with genetically engineered T cells, in the context of both malignant (271) and non-malignant (272) diseases. In the attempt to preserve this early-differentiated T cell subset, the *in vitro* protocol used for TILs expansion was shortened (273) and the anti-CD3/anti-CD28 antibodies were conjugated to cell-size beads (274) or nanomatrixes (275). The cytokine cocktail used to sustain T cell expansion *in vitro* also plays a major role in determining the fitness of cellular products. The introduction of Interleukin-21 in the culture medium promotes a T_CM_ phenotype (276) while Interleukin-7 and Interleukin-15 supplementation, in the absence of IL-2, expands the T_SCM_ pool (277, 278).

As already mentioned, the intrinsic T cell fitness has an impact on the persistence and thus on the efficacy of the T cells used in ACT. In CAR-T cell therapy trials, CAR-T cells isolated from poor responders expressed genes associated with effector memory differentiation and apoptosis, a glycolytic metabolism, and hypo-functionality. Conversely, efficient anti-tumor activity was associated with an early-memory differentiation signature, expression of CD27, and absence of the exhaustion marker PD-1 (259, 279, 280). Similar observations were reported in TCR-based studies. The transfer of a WT1-specific TCR into Epstein-Barr virus-specific donor CD8 T cells has been exploited to generate functional, memory-like cellular products (281). Using this manufacturing procedure, high levels of engraftment and long-term persistence were observed in humans (40). Furthermore, in an ACT trial with TCR engineered T cells, the extent of cytokine release was associated with anti-tumor activity (107).

Once adoptively transferred, T cells interact with the host immune system. Competition of infused and unmodified T cells for proliferative signal accessibility may decrease cell survival. Furthermore, engineered T cells may be recognized and rejected by the host immune system, thus abrogating ACT efficacy. In the autologous setting, rejection could be due to an immune response against the transgene products, as observed in preclinical models (282). An immune response against the murine-derived CD19 CAR was described before and after cell therapy. In the JULIET study (283), the majority of treated patients showed detectable levels of pre-existing anti-murine CD19-specific antibodies, that further increased upon CAR-T cells infusion. Nonetheless, the kinetics of engraftment was unmodified, and rejection barely detected, questioning the relevance of these markers in predicting CAR-T cells persistence. T cell mediated immune responses against Herpes Simplex Virus-derived Thymidine Kinase (TK) epitopes were described in patients treated with TK-DLI, often leading to the elimination of genetically engineered T cells (284). The immunogenicity of TK could be overcome in the HSCT context by infusing transduced T cells during the immunosuppressive phase that follows transplantation (285). For ACT applications that do not involve HSCT, the minimization of transgene immunogenicity remains a desirable and relevant goal.

## Overcoming Barriers to T Cell Homing at Tumor Site

The efficacy of ACT is strictly dependent on the ability of the infused product to infiltrate neoplastic lesions. This is particularly difficult in solid tumors, often characterized by a dense stromal architecture, an abnormal vessel structure, and by alteration of chemo-attractants that impinge T cell homing (286–288). In recent years, different strategies have been developed to counteract these factors and hence, increase the ability of T cells to migrate inside neoplastic lesions, where they can properly exert their anti-tumor activity.

### Interfering With Cancer Metabolism and Chemokines to Increase ACT Infiltration

The connection between metabolism and oncogenesis is well-documented. Metabolic reprogramming, a hallmark of cancer, does not only impact on cancer cell survival and proliferation, but also on the immunological microenvironment. The presence of reactive nitrogen species (RNS), produced by several human tumors, can induce nitration of different proteins present in the tumor microenvironment (TME) with consequences on T cell functions (289). As an example, the nitration of the CCL2 chemokine decreases its binding affinity for CCR2, thus reducing T cell recruitment. In mouse models, preconditioning of the tumor microenvironment with small molecules blocking RNS production increased the CCL2-mediated recruitment of adoptively transferred tumor-specific CD8 T cells (290), making it an interesting target for further therapeutic development.

Tumors can alter the fucosylation of T cell surface glycoproteins (291), again impinging T cell homing at tumor sites. The *ex vivo* glycoprotein fucosylation increases *in vivo* migration and cytotoxic abilities of tumor-specific T cells in leukemia, breast cancer, and melanoma models (292).

The CXCL12/CXCR4 is an additional relevant axis activated by neoplastic cells and cancer-associated fibroblasts in several human tumors. CXCR4 expression correlates with desmoplasia, metastases formation, and immunosuppression (293–298). In murine models of leukemia, melanoma and ovarian cancer, CXCR4 inhibition, obtained with blocking antibodies or with the CXCR4 antagonist AMD3100, increased the effector to T_regs_ ratio at the tumor site and reduced tumor growth (298–301). Stemming from these observations, several clinical trials are now exploring the efficacy of CXCR4 blockade in solid tumors (302).

### Exploiting Cancer Vasculature to Foster ACT Infiltration

Tumor neo-angiogenesis involves the formation of a disorganized network of irregular and leaky vessels, inefficient in delivering oxygen, drugs, and immune cells to the neoplastic microenvironment. This process is largely orchestrated by the vascular-endothelial growth factor (VEGF) and results in tumor growth promotion and altered inflammation (303). VEGF inhibitors are currently used in the treatment of several cancers (304) owing, in particular, to their ability to increase T cell tumor homing (305–309). By promoting vessel maturation, VEGF inhibitors positively impact on immunotherapy and ACT (310). Vascular-targeting peptides represent additional effective tools for the precise delivery of small molecules capable of inducing tumor vessels normalization and increased T cells infiltration. In mouse models, tumor-necrosis factor-targeted (TFN) delivery to the tumor vasculature by linking the TNF protein with the CNGRCG angiogenic vessel-homing peptide (NGR-TNF fusion protein) enhanced the local production of immunomodulatory cytokines, favoring the extravasation of immune cells and improving ACT therapeutic activity (311). The fusion of the TNF superfamily member LIGHT with a vascular targeting peptide (LIGHT-VTP) (312, 313) and the specific delivery of IFN-γ and TNF-α by the homing peptide TCP-1 (314) enhanced endothelial permeability and T cell infiltration in mouse models. The cyclic peptide iRGD (315) and the targeting of the vascular integrity regulator VE-cadherin by CD5-2, a specific inhibitor, facilitated T cell homing to the TME in tumor-bearing mice treated with ACT (316).

Although only some of these therapies have been tested in association with ACT, their effect in increasing T cell migration into tumors strongly suggests that strategies aimed at normalizing the neoplastic vasculature could potentially increase ACT efficacy.

### Enforcing Chemokine Receptor Expression in T Cells

The interaction of specific chemokines and cytokines with their receptors is a key determinant of immune cell migration, and a mismatch between the chemokines secreted by neoplastic or stromal cells and the receptors expressed by T lymphocytes strongly limits T cell homing in tumors (317–320).

In recent years, attempts have been made to correct this mismatch by engineering T cells with receptors for chemokines or cytokines abundant in the TME, showing preliminary encouraging results. In TRAMP mice with metastatic prostate adenocarcinoma expressing high levels of CCL2, the expression of CCR2 in SV40 Tag-specific CD8 lymphocytes increased T cell homing to the tumor (321). In xenograft models, T cell transduction with a RV encoding CX3CR1 enhanced migration toward human cell lines expressing Fractalkine, the CX3CL1 ligand, and inhibited tumor growth (322). In a lymphoma murine model, adoptively transferred CD8 T cells, overexpressing CXCR4, were preferentially recruited by CXCL12 expressing cells in the bone marrow, to promote tumor control (323). Anchoring IL-4 receptors to the membrane of adoptively transferred T cells increased *in vivo* tumor homing, cytokine secretion, and the killing of melanoma (324). Lentiviral T cell transduction to express CXCR2 enhanced *in vivo* homing toward human melanoma in xenograft models, by exploiting the high IL-8/CXCL8 secretion levels (325).

Classical chemo-radiotherapy is also able to modulate T cell recruitment at the tumor site, suggesting that its use with ACT can have additive effects. In murine models, chemo-radiotherapy has been shown to increase the local release of CCL5, CXCL9, and CXCL11 in the TME, improving ACT-induced tumor growth control (326). Treatment with doxorubicin in mouse models bearing either murine or human melanoma induced CXCL9 and CXCL10 expression by neoplastic cells and increased the infiltration of adoptively transferred T lymphocytes. The effect was further enhanced if ACT followed a combined treatment with doxorubicin and IL-2 (327). Unfortunately, the involved chemokine receptor pattern was not deeply investigated, leaving undemonstrated a causal relation between the increased chemokine secretion by tumor cells and the increased T cell homing.

Overall, these studies prompt further investigation and possible exploitation of chemokine receptors in the context of ACT. Interestingly, a large analysis of 142 patients enrolled in ACT trials revealed the association of genetically determined alterations in chemokine receptors expression with response to therapy (328), underlining the knowledge gap on the impact of chemokines in ACT.

### Generating Genetically Engineered T Resident-Memory Cells

Among TILs, T resident-memory (T_RM_) cells are permanent tissue-resident T cells able to mount an immune reaction; they've been proposed to be key determinants in the magnitude of anti-tumor immune responses, and their presence in different human cancers correlates with survival (329–336). It is tempting to assume that the induction and/or manipulation of this T cell subset might improve anti-tumor immunity and disease control. Indeed, in murine models, the administration of anti-cancer vaccines through routes that enhance the induction of T_RM_ is associated to tumor growth inhibition and, importantly, provides protection also at distant sites (330, 337–339). In a melanoma mouse model, ACT with T cells lacking RUNX3, a transcription factor essential for T_RM_ development, strongly reduced TILs accumulation and treatment efficacy (340). These results suggest that the association of ACT with strategies directed at promoting tissue residency of the infused cells might be beneficial to improve tumor control.

## Surviving the Tumor Microenvironment (TME)

**Figure 4:**
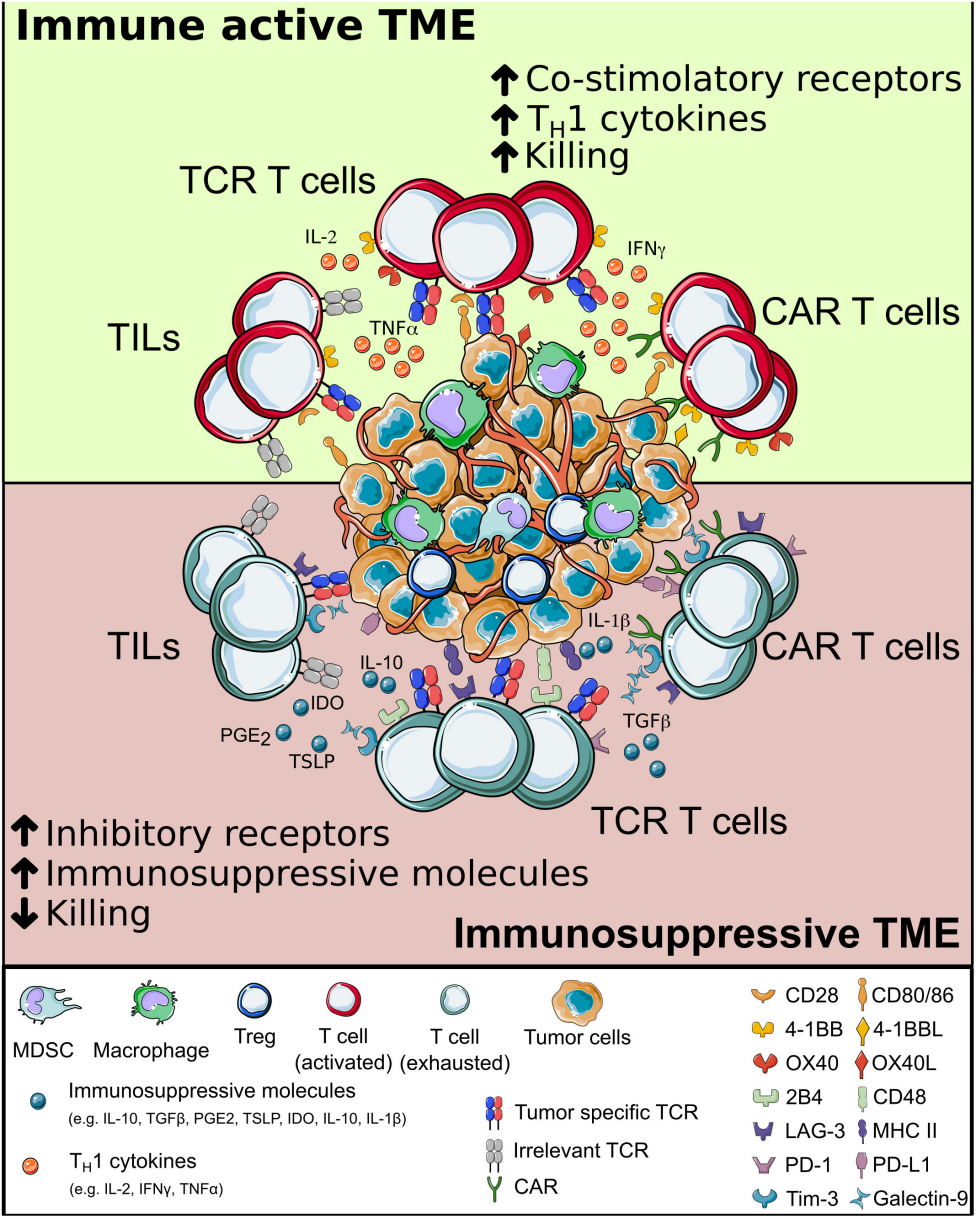
The interplay between T cells and tumor microenvironment. When adoptively transferred T cells (either tumor-infiltrating lymphocytes, Chimeric Antigen Receptor or T cell Receptor-redirected T cells) infiltrate the tumor, they interact with a complex environment, in which a combination of intracellular signals compete. When inflammatory signals dominate, T cells can perform effector functions and potentially eradicate cancer cells; else, they may become exhausted, have limited survival and fail in killing tumor cells. CAR, chimeric antigen receptor; TCR, T cell receptor.

As already demonstrated in several disease contexts, a long-lasting protective memory is a critical requirement for a successful ACT. Despite improvement in manufacturing protocols and fitness of the final cellular products, adoptively transferred T cells have to face a harsh environment while interacting with cancer cells (Figure 4). Different immunosuppressive mechanisms act at the tumor site, with the potential to reduce or even dampen the curative action of ACT. If a T cell encounters the cognate antigen in an immune active environment with plenty of additional co-stimuli, the resultant immune reaction is most likely to be efficient in clearing antigen-bearing cells. However, at the tumor site a variety of signals make T cells chronically exposed to antigenic stimulation in the absence of appropriate co-stimuli. Different TME resident cells and tumor cells are responsible for this, either by directly interacting with T cells or by releasing soluble factors. As a consequence, T cell depletion, anergy, exhaustion, and accumulation of T_regs_ (341–345) promote tumor immune escape (214).

### Checkpoint Blockade and ACT

Tumor-specific T cells infused to patients and chronically exposed to tumor antigens often enter a dysfunctional state defined as T cell exhaustion that hampers the anti-tumor response and fosters tumor escape (346).

T cell exhaustion is a process of progressive and hierarchical loss of effector functions (e.g., chemokines production and cytolytic activities), resistance to activation through TCR engagement, metabolic deregulation, and failure to acquire an antigen-independent memory state (347). The sustained co-expression of multiple inhibitory receptors (IRs) such as PD-1, CTLA-4, LAG-3, Tim-3, 2B4, CD39, CD160, BTLA, and TIGIT was identified as a hallmark of exhausted T cells (T_EX_) (249) in solid and hematological tumors (e.g., melanoma, leukemia, breast, prostate, ovarian, renal, lung, and hepatocellular carcinoma (348–358). Interestingly, the pattern of inhibitory receptors expressed by T_EX_ significantly varies among different tumor types. This indicates that exhaustion mechanisms are differentially shaped by various tumor microenvironments (248) and suggests that ACT approaches need to be tailored according to the specific features of each tumor.

Blocking the interactions between the IRs expressed by tumor-reactive T cells and their cognate ligands leads to the reversal of T cell exhaustion (359, 360). Monoclonal antibodies impeding the PD-1/PD-L1 binding (361) and affecting the CTLA-4 axis (362, 363) are able to restore tumor T cell recognition and tumor regression in a relevant subset of terminally ill patients. Due to their efficacy, the use of monoclonal antibodies in association with ACT could greatly benefit T cell resistance toward the tumor microenvironment. Although still exploratory, this combinatory approach showed encouraging preclinical results in the context of CAR-T cells (364).

The use of an immune checkpoint blockade is associated with significant toxicity, indicating that the indiscriminate blockage of inhibitory receptors on the entire T cell repertoire may be deleterious. Indeed, immune-related adverse events (irAEs) occurred in up to 80% of treated patients, and were life threatening in a significant fraction of cases (365). irAEs mainly occurred because potential autoreactive T cells were unleashed and T_regs_ functionality was dampened (366). In order to maintain the benefit of inhibitory receptor blockade while reducing toxicities, it may be beneficial to counteract inhibitory axes selectively on tumor-specific T cells. This is currently one of the focuses in the T cell-based immunotherapy field. The increased anti-tumor activity of CAR- and TCR-engineered T cells in which an inhibitory receptor gene has been disrupted was shown in different preclinical models (161, 166, 171, 367). Recently, the results of the first-in-human phase I trial with PD-1 disrupted TCR-edited autologous T cells in patients with refractory tumors have been published, highlighting the feasibility and safety of multiplex gene-editing in tumor-specific T cells (42).

T cell exhaustion can also be exploited for tumor-reactive T cell isolation purposes. In fact, in the context of melanoma, it was shown that PD-1 expressing TILs are enriched in melanoma-specific T cells. These cells, despite being exhausted, could be isolated and their function restored (224). In addition, circulating PD-1 positive T cells showed to be enriched in neoantigen-specificities (368) when compared to the PD-1 negative counterparts.

### Counteracting Immune Suppressive Cells Accumulating in TME

Besides T cell exhaustion, the presence of immunosuppressive cell subpopulations or soluble cytokines may also dampen anti-tumor T cells responses. In tumors, monocytes have been described to preferentially polarize into M2-tumor associated macrophages (M2-TAM) (369) or Tie-2-expressing monocytes (TEM) (370). M2-TAM and TEM sustain tumor survival and blunt immune reactions. Myeloid-derived suppressor cells (371) and different components of the stroma have been implicated as well in tumor progression, through different mechanisms: (i) the expression of inhibitory receptor ligands (372), (ii) the production of metabolites or soluble factors [e.g., indoleamine 2,3-dioxygenase (IDO) (373), Interleukin-1β and thymic-stromal lymphopoietin (TSLP) (374, 375), and prostaglandin E2 (376)], or (iii) alteration of pH and oxygen levels (377–380). Among the soluble cytokines, the role of TGF-β gained particular attention. TGF-β is released by neoplastic cells of different origins (381) and its secretion is linked to common cancer genetic mutations (382). At the tumor site, TGF-β acts as a local immunosuppressor, thus reducing the effect of immunotherapy on cancer cell growth. In ACT models, the infusion of CD8 T cells genetically manipulated to resist TGF-β outperformed TGF-β sensitive cells in mediating tumor control (383–387). Interestingly, in a recent clinical study 4 out of 8 patients with Hodgkin lymphoma treated with tumor-specific T cells engineered to express a dominant negative form of TGF-β receptor type II (388) experienced an objective clinical response (389).

To help engineered T cells in counteracting the immunosuppressive microenvironment, different strategies are currently under scrutiny. CAR-T cells have been genetically modified to secrete Interleukin-12 or Interleukin-18 (TRUCK cells) upon CAR engagement (390). A deeper understanding of the expression profile associated to functional CAR-T cells has been now translated in new manipulation processes, involving the overexpression of transcription factors, such as c-Jun (391), or the deletion of inhibitory molecules such as REGNASE-1 (392). These newly proposed strategies could lead to the generation of T cell products endowed with early differentiated phenotypes and enhanced anti-tumor functionality.

## Final Remarks

Adoptive T cell therapy represents a unique and innovative therapeutic pillar for cancer treatment. T cells couple the ability to circulate and home at different sites, to sense and respond to the surrounding environment and to persist long-term, thus providing immunosurveillance against residual malignant cells. Each of these characteristics, intrinsic to T cell biology, is however challenged by several immune escape mechanisms active in cancer patients.

Table 1 summarizes the efficacy and toxicity profiles of TCR-redirected T cells reported in clinical trials. Results demonstrate the feasibility of the approach, indicating its therapeutic potential, but also underline the challenges that TCR-based ACT needs to face. Firstly, a suboptimal efficacy of TCR-based studies has currently been observed in patients and no clinical results have been published yet with engineered T cells targeting neoantigens or MiHA. Both evidences underline the difficulty in isolating high affinity tumor-specific TCRs that might be exploited for treating a large number of patients. Secondly, extensive *in vitro* and *in vivo* assays are necessary to lower the incidence of adverse events and increase the safety profile of the infused T cell products.

An interesting point of discussion is whether CAR-T cell therapy should be preferred to TCR-based T cell therapy or vice versa. The answer likely lies in the middle and might envisage the alternated or even the combined use of TCR- and CAR-T cells in different clinical settings, according to the antigenic profile of each tumor type. Combinations could exploit the strength of both strategies. Despite CAR-T cells can count on the extensive level of knowledge acquired on cancer cell phenotypes, testable targets remain few. It's possible that this effect underlies the intrinsic limitation of CAR-T cells, able to target surface proteins but at present unable to recognize all the intracellular mutated or overexpressed proteins in cancer cells. An engineered TCR, instead, has the ability to virtually recognize every tumor antigen, independently of intracellular localization, including mutated molecules, intracytoplasmic proteins, and transcription factors. Hence, finding suitable targets for TCR-engineered T cells is theoretically easier. However, the HLA-restriction of TCR-based immunotherapies needs to be taken into consideration.

In terms of efficacy, CAR-T cells showed optimal results in the context of relapsed/refractory ALL. TCR therapy seems promising in liquid tumors, but both CAR and TCR-engineered T cell therapies showed less than satisfactory results in solid tumors other than melanoma when compared to TILs therapy.

In terms of signaling, TCRs are sensitive to much smaller epitope densities than CARs (393) and T cell activation is finely tuned by the affinity and the avidity for the ligand itself. These differences may prove important when dealing with low antigen density and also in promoting immunological memory while avoiding T cell exhaustion. Even if the precise role of the different signals conveyed in the immunological synapse are still not completely understood, TCR signaling might be more rewarding in the ability to balance T cell activation, possibly solving the limitation in T cell persistence and functionality reported in ACT with CARs.

The possibility of genetically engineering T cells by redirecting their specificity toward cancer by employing CARs or TCRs has already produced relevant clinical results in patients affected by selected tumor types. By combining T cell therapy with alternative therapeutic approaches (i.e., checkpoint blockades) and by implementing multiple genetic manipulation (i.e., by genome editing) in T cells, the efficacy of cancer immunotherapy is further increasing, and we might envisage its successful extension to a larger range of tumor types. As the field progresses, several challenges, including manufacturing complexity, regulatory issues, and sustainability will need to be faced, with the ultimate aim of offering this new therapeutic tool to all patients who could benefit.

## Author Contributions

AP, BC, ET, ER, FM, and MN did the primary research and wrote the manuscript. AP, BC, and FM edited the figures. FM and ER oversaw the preparation of the manuscript. FC, AB, CB, and ER edited the final draft. All authors contributed to the article and approved the submitted version.

## Conflict of Interest

The authors declare that the research was conducted in the absence of any commercial or financial relationships that could be construed as a potential conflict of interest.
